# Non-apoptotic role of caspase enzymes in satellite cells from human skeletal muscle

**DOI:** 10.1007/s10522-026-10478-1

**Published:** 2026-07-25

**Authors:** Roberta Di Pietro, Rosa Mancinelli, Gianna Impicciatore, Gianmarco Stati, Stefania Fulle, Silvia Sancilio

**Affiliations:** 1 Department of Medicine and Aging Sciences, “G. d’’Annunzio” University of Chieti-Pescara, Via dei Vestini 31, 66100 Chieti, Italy; 2 StemTeCh Group, CAST, “G. d’’Annunzio”” University of Chieti-Pescara, Via Luigi Polacchi 11, 66100 Chieti, Italy; 3 Center for Biotechnology, Department of Biology, College of Science and Technology, Sbarro Institute for Cancer Research and Molecular Medicine, Temple University, Philadelphia, PA 19122 USA; 4 Department of Neuroscience Imaging and Clinical Sciences, “G. d’Annunzio” University of Chieti-Pescara, Via dei Vestini 31, 66100 Chieti, Italy; 5 UdA-TechLab, Research Center, “G. d’Annunzio” University of Chieti-Pescara, Via dei Vestini 31, 66100 Chieti, Italy

**Keywords:** Muscle stem cells, Aging, Stress conditions, Apoptosis, Differentiation

## Abstract

Satellite cells (SCs) are essential for skeletal muscle regeneration, but their function declines with aging, often associated with increased pro-apoptotic signaling. This study investigated the impact of in vitro serum starvation—as a model of acute microenvironmental and nutrient stress—on the apoptosis and differentiation potential of human SCs from young and aged donors. SCs were isolated from the Vastus Lateralis of young and aged subjects and cultured in serum-free medium for up to 72 h. We assessed apoptosis through Annexin V/PI staining, TUNEL assays, and caspase activity measurements, while transcriptional profiles were analyzed via RT-PCR. Aged SCs displayed a significantly higher susceptibility to stress-induced apoptosis compared to young controls, marked by the early upregulation of CASP9 and FOXO1. While typical nucleosomal DNA fragmentation was absent, we observed the activation of caspase-3 after 72 h of starvation. In aged cells, activated caspase-3 co-localized with myogenin and extranuclear DNA at sites of nuclear remodeling. Notably, treatment with a pan-caspase inhibitor (z-VAD-fmk) prevented the formation of micronuclei and myotubes, further highlighting a non-apoptotic role for these enzymes. Aged SCs also showed a distinct cell cycle profile characterized by an enlarged G0/G1 phase and altered expression of CDK and CCNB1 genes. Our findings suggest that in human aged SCs, caspase enzymes serve a dual role: mediating a heightened stress response and facilitating the nuclear remodeling necessary for myogenic differentiation. These results clarify how intrinsic aging shapes the response of muscle stem cells under severe environmental and metabolic resource deprivation.

## Introduction

Skeletal muscle is composed of long multinucleate and contractile structures: muscle fibres, which during myogenesis originate from the fusion of myoblasts into multinucleate myotubes (Pietrangelo et al. 2009; Panda et al. 2016). Myofibres regeneration, during muscle repair, relies on its resident stem cells: SCs (García-Prat et al. 2016). These adult stem cells are localized between the basal lamina and the plasma membrane of muscle cells. SCs are usually quiescent in adult muscles. After a muscle lesion, they are induced to proliferate, migrate to the site of lesion and merge with existing myofibres or to generate new fibres (Fulle et al. 2013; García-Prat et al. 2016). A small fraction of activated SCs can return quiescent for self-renewal (Dumont et al. 2015).

Aging is an elaborated process that, in pluricellular organisms, manifests as the result from the interaction among cells, inter-cellular communication and systemic dysregulations, all of which, in a co-ordinated manner, impair the homeostatic capacity of the organism. Aging of SCs is characterized by a reduction in stem cells amount and capability, which has been ascribed to several factors, such as errors in self-renewing mechanisms, loss of differentiation ability, apoptosis, and senescence (G0 irreversible arrest state) (Stefania Fulle et al. 2012; Sousa-Victor and Muñoz-Cánoves 2016). The main implication of SC aging is the decline in the capacity of muscle regenerative potential and the gradual atrophy of skeletal muscle. This status, called sarcopenia, has significant health care consequences for humans, for its contribution to fragility, functional loss, and early cell death (Fulle et al. 2013). Despite the enormous number of investigations, there is an intensive discussion in the scientific community to clarify how age-related inefficient muscle regeneration is driven by modifications in extrinsic environment, that are able to inhibit the regenerative capability of diversely competent SCs, or by an age-related degradation of SC role due to an intrinsic aging of the resident stem cells, which make them less responsive to environmental stimuli, or indeed by a combination of both conditions (Stefania Fulle et al. 2012; Brack and Muñoz-Cánoves 2015).

In previous works, we showed an age-related reduction in the antioxidant ability of human SCs that may adversely influence the aging SC ability to repair muscle (Fulle et al. 2005; Beccafico et al. 2007). More recently, we showed the occurrence of spontaneous apoptosis in aged SCs cultured in vitro, supporting the hypothesis of an intrinsic aging of human SCs (Fulle et al. 2013). However, how these cells respond to severe environment-induced stress remains largely unexplored. While spontaneous apoptosis reflects basal fitness, starvation-induced stress triggers distinct survival and differentiation networks. In this study, we aimed to investigate the effects of serum and nutrient deprivation on apoptosis and/or differentiation of aged SCs compared with younger counterparts. While we acknowledge that serum starvation cannot serve as an absolute surrogate for the complex pathophysiology of in vivo ischemia due to confounding metabolic responses (Pirkmajer and Chibalin 2011), this severe starvation medium (SM) provides a robust in vitro model to explore how satellite cells cope with extreme environmental stress, characterized by the localized growth factor and nutrient withdrawal that typically occurs during tissue injury or compromised vascular supply (Pirkmajer and Chibalin 2011). The role of both initiator caspases (caspase-8 and −9), and effector caspases (caspase-3) was analysed in the presence or absence of specific or broad pharmacologic inhibitors to better understand the role of apoptosis in SCs death or differentiation. To assess the mechanisms related to these processes we evaluated the expression of multiple genes involved in mediating proliferation, differentiation, oxidative stress, inflammation, atrophy, and ubiquitination.

## Materials and methods

### Subjects, SCs culture and treatments

SCs cultures derived from human donors were obtained, and inclusion/exclusion criteria applied, as previously described (Fulle et al. 2013) from twelve healthy male young subjects (23.8 ± 2.5 years old; ‘young SCs’) and nine healthy male old subjects (72.3 ± 1.5 years old; ‘aged SCs’, see Table 1) after written informed consent and approval from the ethics committee of the ‘G. d′Annunzio’ University of Chieti–Pescara (protocol numbers: 1233/06 COET, 25/July/2006; 1884 COET, 15/May/2009; and 1634/08 COET, 24/June/2008). About 16 h after plating, the Growth Medium (GM) was replaced with the SM made of HAM’s F-10 enriched with 50 μg/mL gentamycin and 0.1% bovine serum albumin (BSA). Experiments were performed on young and aged SCs after 4–24–48 and 72 h of culture in SM (15.7 PDL for young SCs and 12.2 PDL for aged SCs). At each time point, cells were pre-treated for 45 min with the specific caspase-8 (20 μm z-IE(OMe)TD(OMe)-fmk), caspase-9 (50 µm z-LE(OMe)HD(OMe)-fmk) or broad-spectrum z-VAD-fmk (20 μm) caspases inhibitors. All reagents were from Calbiochem (La Jolla, CA).

**Table 1:** Donor demographics and clinical characteristics

Characteristic	Young SCs (n = 12)	Aged SCs (n = 9)
Age (years, mean ± SD)	23.8 ± 2.5	72.3 ± 1.5
Sex (Male/Female)	*12 MALE*	*9 MALE*
Health Status	Healthy (as per Fulle et al. 2013)	Healthy (as per Fulle et al. 2013)

### Annexin V-propidium iodide detection in flow cytometry

Early apoptotic cells were identified through reversible Annexin V binding to phosphatidylserine, a membrane phospholipid exposed early in apoptosis (Fadok et al. 1992). Apoptosis was assed using a commercial human Annexin V-FITC kit (Bender MedSystem, Vienna, Austria) as previously reported (Caravatta et al. 2008). Analyses were performed with an EPICS Coulter flow cytometer (FL3 detector, a log mode), using EXPO 32 analysis software (Beckman Coulter Inc., Brea, CA). For each sample, 10.000–20.000 events were collected. Vital cells were Annexin V-/PI-, early apoptotic cells Annexin V +/PI−, late apoptotic cells Annexin V +/PI + and necrotic cells were Annexin V−/PI +.

### DNA analysis for flow cytometry

For each experimental condition 25 × 104 cells were collected, fixed, and maintained overnight at 4 °C. Cells were then resuspended in 20 µg/mL PI and 100 µg/mL RNAse, final concentration. Cell cycle profiles were evaluated with an EPICS-XL cytometer using the EXPO32 software (Beckman Coulter, Inc.) as previously reported (Caravatta et al. 2008). Cells with low fluorescence indicated low DNA content were gated as dead cells. Data were analysed using Multicycle software (Phoenix Flow Systems, San Francisco, CA, USA).

### Immunofluorescent staining of DNA strand breaks

DNA damage was assessed via TUNEL assay, using the in-situ Cell Death detection kit (Boehringer Mannheim, Mannheim, Germany) as previously reported (Ansari et al. 1993; Giampietro et al. 2006). For digital imaging, cells cultured as a flat monolayer on coverslips were counterstained with DAPI mounting medium (Vector Laboratories, Newark, CA) and observed with a ZEISS Axioskop 40 (Carl Zeiss, Göttingen, Germany) widefield light microscope. Images were acquired using the 20 × (NA = 0.45) and 40 × (NA = 0.65) objective lenses, and captured via a Coolsnap videocamera (Photometrics, Tucson, AZ). DNA fragmentation was evaluated by counting green-fluorescent nuclei at 40 × magnification. Five slides per sample were examined scoring apoptotic cells out of 100. Positive control were SCs treated with 3 µL deoxyribonuclease I for 10 min at RT.

### Measurement of caspase activation

To analyse caspase activation, two assays were used. Caspase-8 activation was assessed using the FLICA Apoptosis Detection kit (Immunochemistry Technologies, Bloomington, MN, USA) based on a fluorochrome inhibitor of caspases (Ekert et al. 1999), as previously described (4). Samples were examined employing an EPICS Coulter flow cytometer (FL3 detector, log mode) using EXPO 32 analysis software. Caspase-3 activation was evaluated by immunofluorescence using a rabbit monoclonal anti cleaved caspase-3 (1:200; #9664, Cell Signalling Technology), followed by goat anti-rabbit IgG TRITC (1:50; #111-025-003, Jackson Immuno Research) diluted. Slides were then TUNEL processed, counterstained with DAPI (Vector Laboratories), and observed under light microscopy. To assay myogenin and cleaved caspase-3 expression, young and aged SCs were stained by double indirect immunofluorescence, using mouse anti-myogenin monoclonal antibody (1:50; #sc-12732, Santa Cruz) and a rabbit anti-cleaved caspase-3 antibody (1:100; #9579, Cell Signalling), followed by goat anti-mouse IgG TRITC and goat anti rabbit IgG FITC (1:50; #111-025-003, Jackson Immuno Research).

### Real time-PCR

SCs were cultured for about 16 h in GM. RT-PCR was done on young and aged SCs at 4–24 to 48–72 h in SM. Total RNA was isolated using the standard TRIzol protocol (Sigma-Aldrich, Milan, Italy) and checked for quantity and quality. To account for inter-individual biological variability among primary human donors, equal amounts of cDNA derived from three (n = 3) independent donors per group were pooled together for each time point. TaqMan Low Density Array (Applied Biosystems-MDS Sciex, Toronto, Canada) was used as a single, definitive high-throughput screening tool was used for relative RNA quantification as previously described (Fulle et al. 2013). This specific microfluidic card configuration allowed the simultaneous screening of a panel of 48 target genes per condition in a single run. Data collected using SDS software were used to calculate target gene expression following the arithmetical formula 2–DDCt, using GAPDH as endogenous control.

### Statistical analysis

All quantitative data are reported as mean ± SD. Statistical analyses were performed using the one-way ANOVA or one-tailed Student t-test. Values of p < 0.05 were considered statistically significant.

## Results

### Aging makes SCs more prone to stress-induced apoptosis

**Fig. 1:**
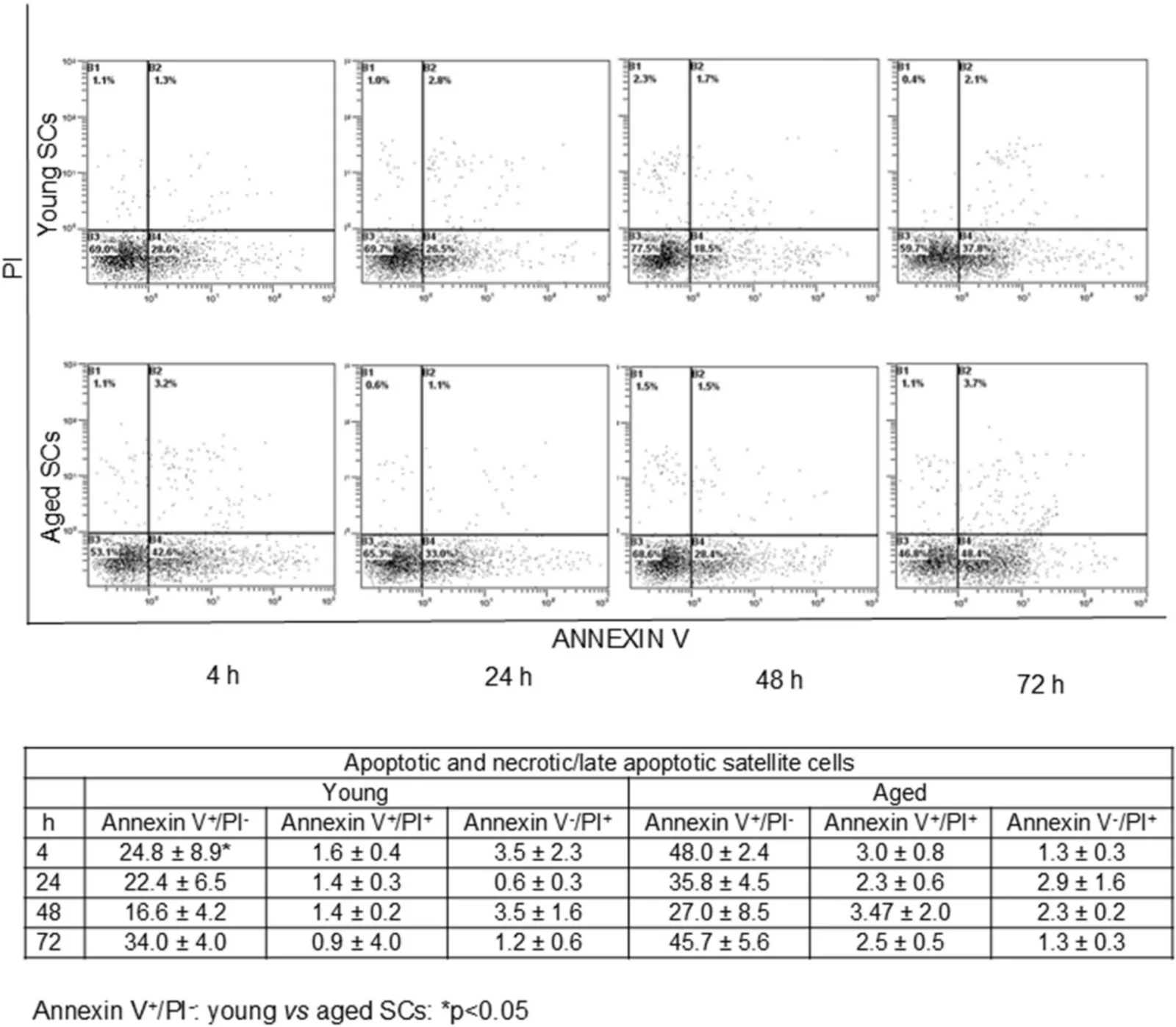
Annexin V/PI detection in flow cytometry. Flow cytometry dot plots of different cell populations in young SCs and aged SCs cultured for 4–24–48 and 72 h, as indicated. Early apoptotic cells (AnnexinV +/PI− quadrant B4) can be discriminated from viable cells (AnnexinV-/PI−, quadrant B3), late apoptotic cells (AnnexinV +/PI +, quadrant B2), and necrotic cells (AnnexinV−/PI +, quadrant B1), according to their fluorescence emission. The most representative out of four separate experiments is shown. Annexin V +/PI−: young versus aged SCs: *p < 0.05. Apoptotic and necrotic/late apoptotic cell values are presented in the lower section of the figure

To evaluate any age-related changes in SCs susceptibility to apoptosis, we initially employed flow cytometry to quantify the percentage of Annexin V^+^ SCs after 4–72 h in culture under starvation. As shown in Fig. 1, the percentage of apoptotic SCs was significantly (p < 0.05) higher in aged subjects (‘aged SCs’) compared to young controls (‘young SCs’) at 4 h, though it was increased in aged SCs at all experimental time points.

**Fig. 2:**
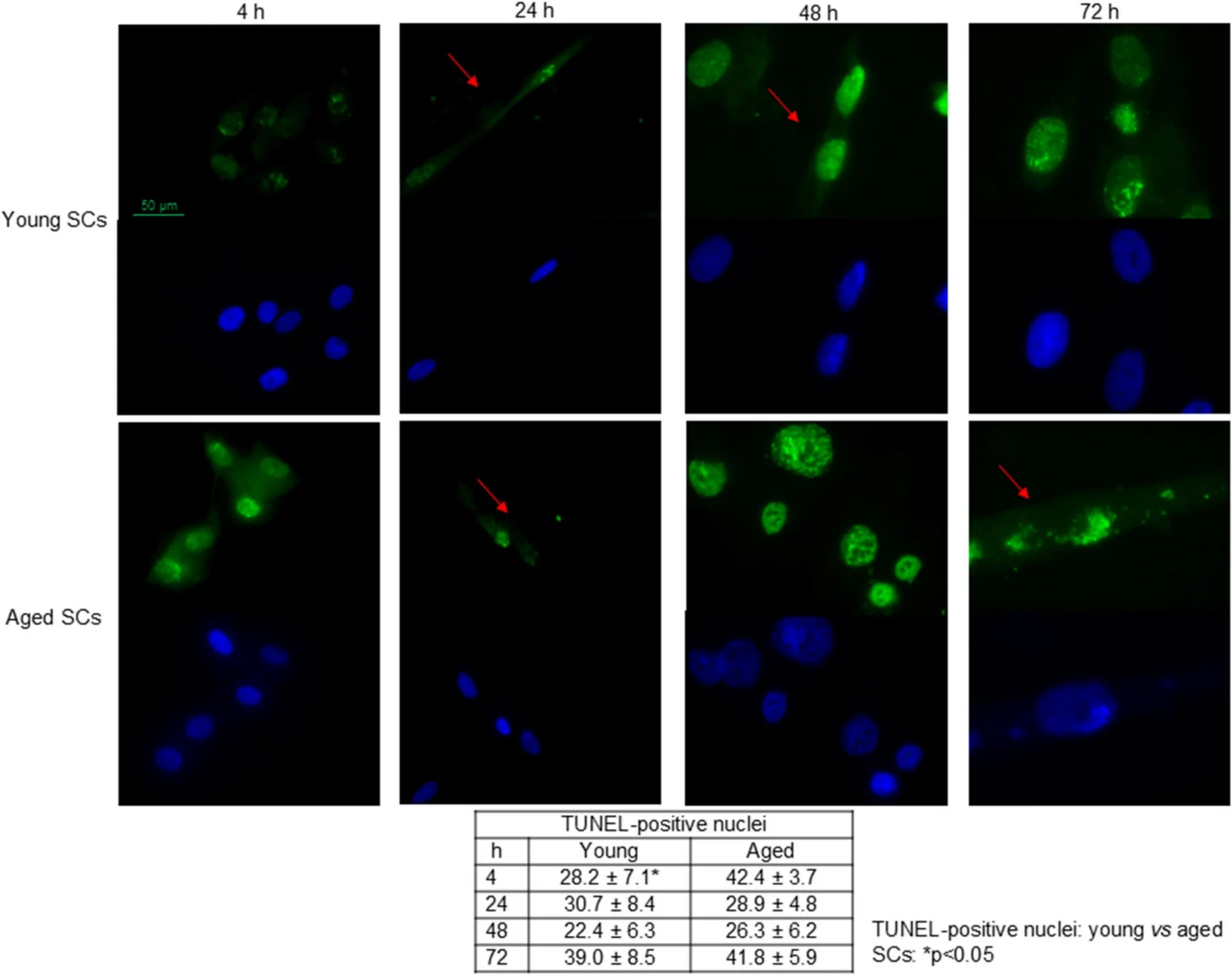
Detection of DNA strand breaks. Fluorescence images of young SCs and aged SCs assayed with the TUNEL technique at different time intervals of in vitro culture, as indicated. Nuclei were counterstained with DAPI (blue fluorescence). Green (TUNEL) and blue fluorescence (DAPI) single emissions are shown in the top and bottom panels, respectively. Red arrows indicate the presence of small myotubes. Representative fields from a representative experiment of the three independent experiments are shown. Original magnification: 40 ×. Scale bar: 50 µm. The counts of TUNEL-positive nuclei are presented in the lower part of the figure

Thus, this significant increase was independent on the culture duration since the number of Annexin V^+^ SCs in the elderly doubled the young control percentage after only 4 h in culture (48.0 ± 2.4 *vs* 24.8 ± 8.9, p < 0.05). Interestingly, the detection of DNA strand breaks with the TUNEL highlighted positive nuclei inside newly formed myotubes both in aged and in young subjects (Fig. 2). TUNEL positive cells showed the typical nuclear labelling, and, in aged SCs, an atypical dot-like pattern of labelling finely dispersed in the cytoplasm especially noticeable after 48–72 h in culture. Moreover, the number of positive nuclei showed a significant difference between aged and young groups only at the 4-h (42.4 ± 3.7 vs 28.2 ± 7.1). Furthermore, several aged SCs displayed an elongated shape and a greater size than the younger counterparts after 72 h culture under starvation (Fig. 2).

**Fig. 3:**
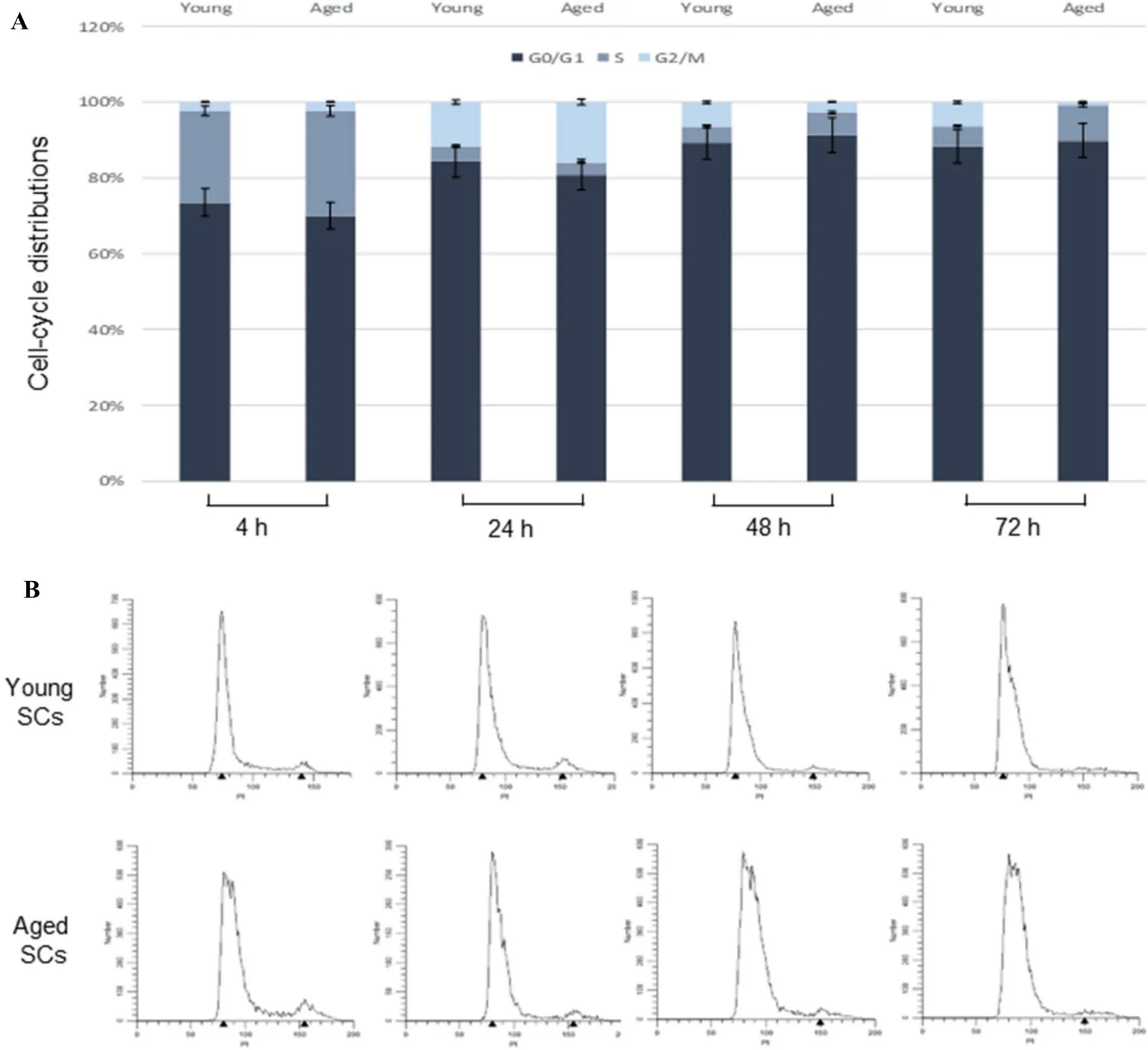
Cell cycle evaluation in flow cytometry. **A** Cell cycle distributions of young SCs and aged SCs at different time intervals of in vitro culture, as indicated. Data are the mean ± S.E. of three independent experiments. Significant differences are seen for aged SCs versus young SCs: for G0/G1 at 24 h (p < 0.05); for S at 24–48 and 72 h (p < 0.05); for G2/M at 72 h (p < 0.05) **B** Cell cycle profiles of young SCs and aged SCs at 4–24–48 and 72 h of in vitro culture, as indicated, showing the absence of any hypodiploid peak. A representative experiment of three independent experiments is shown

This was consistent with the raise in the S phase (at 4–48–72 h) and the decrease in the G_2_/M phase (48–72 h) of the cell cycle in the elderly in comparison to young controls that, in turn, displayed a decrease in the S phase (4–48–72 h) and in the G_0_/G_1_ phase at 48–72 h, and an increase in the G_2_/M phase at 48–72 h (Fig. 3A). Surprisingly, whatever was the duration of the culture, no hypodiploid peak (sub G_0_/G_1_ cell population) was detected in cell cycle profiles of aged SCs (Fig. 3B). This finding can be justified either by the absence of the typical DNA fragmentation at the nucleosomal level or by the incomplete execution of the apoptotic program.

### The caspase enzymes have dual roles in stress-induced SCs cell death from aged and young subjects

**Fig. 4:**
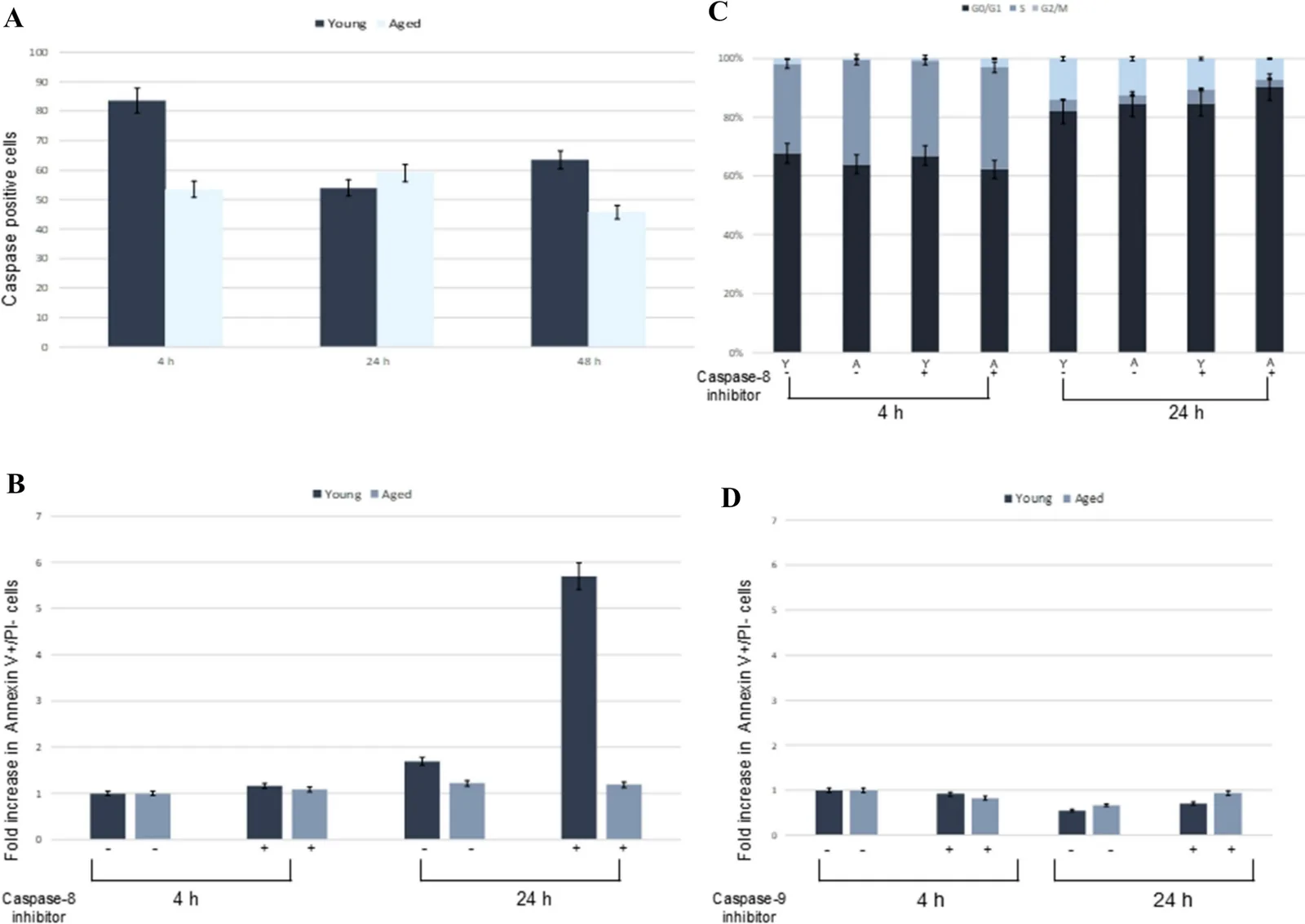
Caspase-8 activation analysis. **A** Caspase-8 activation in young SCs and aged SCs at different time intervals of in vitro culture, as indicated. Data are the mean ± S.E. of three independent experiments. A significant difference is seen for aged SCs versus young SCs at 24 h (p < 0.01). **B** Fold-increase in AnnexinV +/PI- young SCs and aged SCs prior to and after administration of a caspase-8-specific inhibitor, as indicated. The data were obtained at 4 and 24 h and were normalised to the AnnexinV +/PI- cell levels without the inhibitor, as the mean ± S.E. of three independent experiments. A significant difference is seen for aged SCs versus young SCs at 24 h (p < 0.05). **C** Cell cycle distributions of young SCs and aged SCs at different time intervals of in vitro culture prior to and after administration of a caspase-8-specific inhibitor, as indicated. A representative experiment of three independent experiments is shown. **D** Fold-increase in AnnexinV +/PI- young SCs and aged SCs prior to and after administration of a caspase-9-specific inhibitor. The data were obtained at 4 and 24 h and were normalised to the AnnexinV +/PI- cell levels without the inhibitor, as the mean ± S.E. of three independent experiments. A significant difference is seen for aged SCs versus young SCs at 24 h (p < 0.05)

To assess whether caspase enzymes were associated with stress-induced cell death of SCs, we considered the expression and activity of the initiator caspases that are responsible for extrinsic (caspase-8) and intrinsic (caspase-9) apoptotic pathway recruitment. Remarkably, caspase-8 activity was found higher in young SCs at 4–48 h (Fig. 4A). To assess the involvement of caspase-8 in mediating SCs apoptosis under starvation, we treated cells with a specific caspase-8 inhibitor and assayed Annexin V/PI expression in flow cytometry. Indeed, after in vitro treatment with a caspase-8 specific pharmacologic inhibitor, the percentage of Annexin V +/PI− cells significantly increased at 24 h in the young in comparison with the elderly (Fig. 4B). This different behavior between the age-groups under study was observed also in the cell cycle profile which showed an increased S phase in the young at 24 h after treatment with the caspase-8 specific pharmacologic inhibitor, whereas in aged SCs the G0/G1 phase was enlarged at the same experimental time point (Fig. 4C). Unexpectedly, the percentage of Annexin V +/PI−- SCs from both young and aged subjects did not differ significantly after 4–24 h treatment in culture with a caspase-9 specific pharmacologic inhibitor (Fig. 4D).

**Fig. 5:**
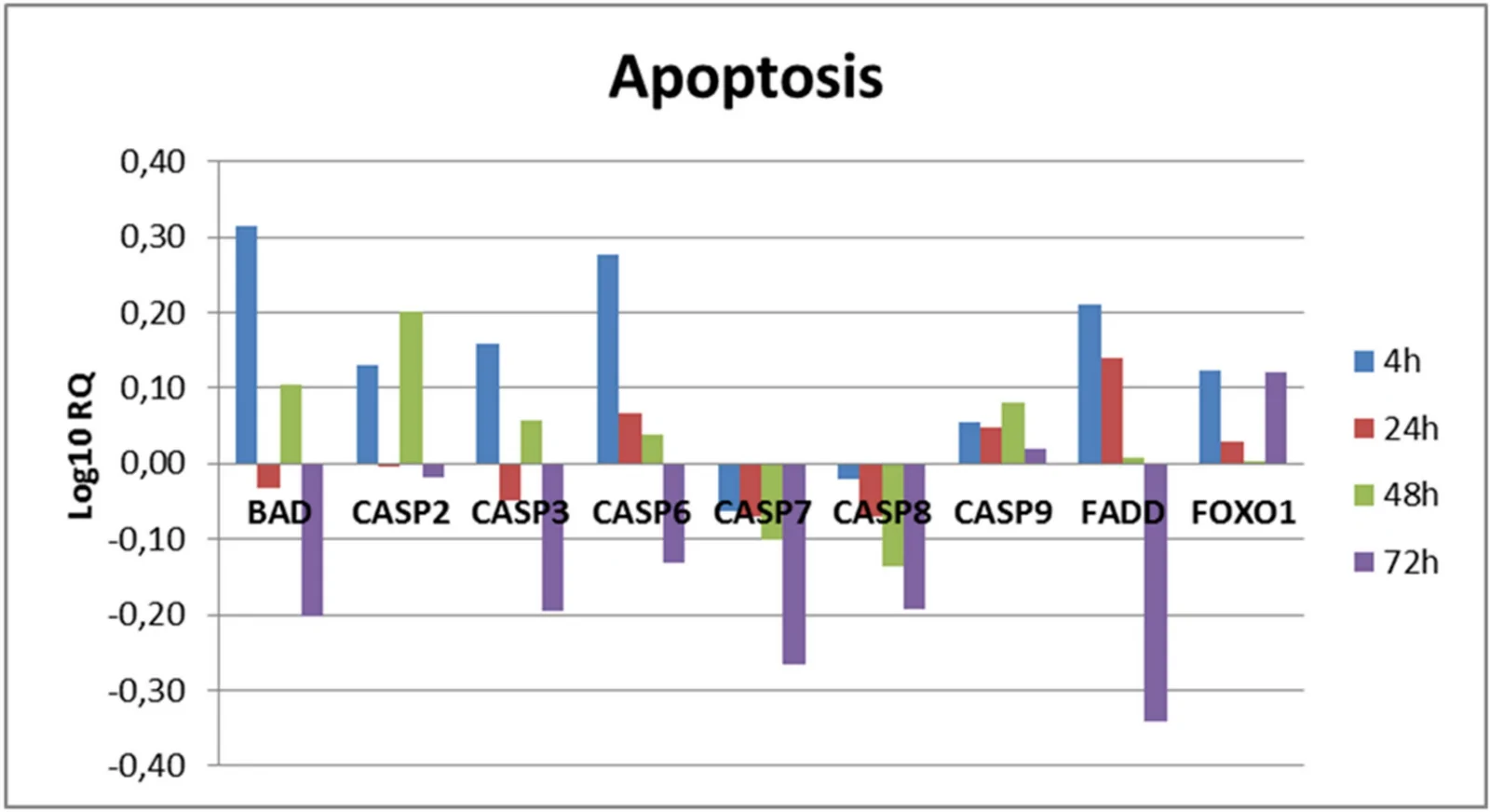
Evaluation of genes involved in the apoptosis pathway. Expression levels of genes involved in the apoptosis pathway analysed with RT-PCR using TaqMan low density arrays. BAD, CASP2, CASP3, CASP6, CASP7, CASP8, CASP9, FADD and FOXO1 mRNA expression levels in aged SCs versus young SCs in SM for 4, 24, 48 and 72 h. Data are expressed as Log10 of Relative Quantification of transcripts for these target genes, each versus GAPDH gene expression. To account for inter-individual donor variability, cDNA samples from three (n = 3) independent biological donors per group were pooled and analyzed in a single definitive run

Concerning the expression of apoptotic genes in starvation medium (SM), it was noteworthy that for all the caspase genes investigated (except for CASP7 and CASP8), and for BAD and CASP6, there was an early upregulation in the aged SCs (Fig. 5). Also of note, CASP9 was upregulated at all time points tested. Instead, CASP6 and FADD, which were upregulated at 4–24−48 h, became downregulated at 72 h. Furthermore, whereas FOXO1 was upregulated at all time points tested (except at 48 h as it was not dysregulated), BAD, CASP2 and CASP3 were upregulated at 4–48 h and downregulated at 24–72 h (Fig. 5).

**Fig. 6:**
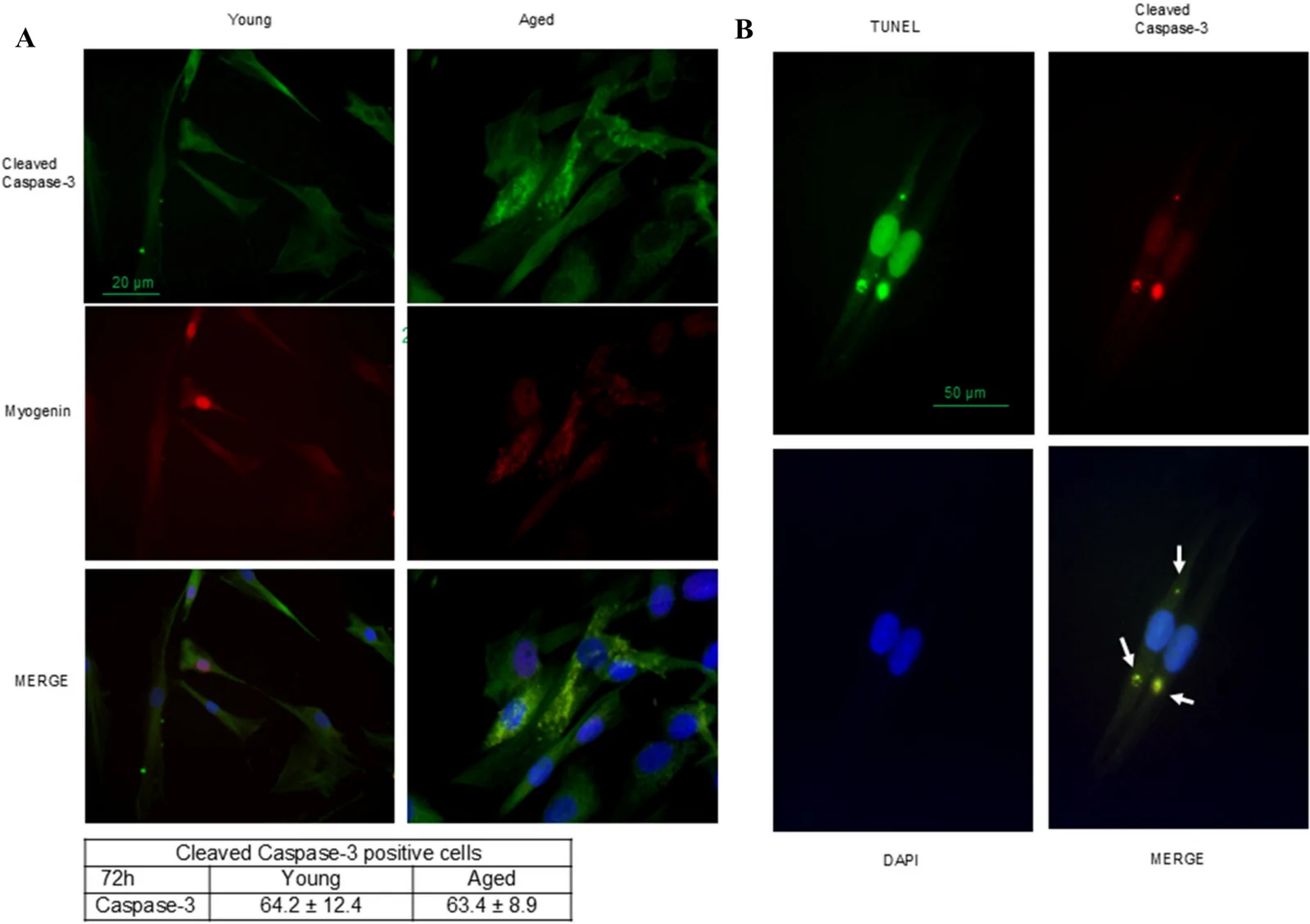
Caspase-3 activation and myogenin expression. **A** Immunofluorescence images of cleaved caspase-3/myogenin detection in young SCs (left) and aged SCs (right) following in vitro culture for 72 h. Green (cleaved caspase-3), red (myogenin), and blue (DAPI) fluorescence single emissions are shown, as indicated. Of note, the nuclear and cytoplasmic location of caspase-3 is appreciable only in the aged SCs, while the nuclear localisation of myogenin is visible in young SCs. Representative fields from a representative experiment of three independent experiments is shown. Magnification: 20. Scale bar: 20 µm. The quantification of caspase-3–positive cells cell values is presented in the lower section of the figure **B** Immunofluorescence images of cleaved caspase-3/TUNEL detection in aged SCs following in vitro culture for 72 h. Green (TUNEL), red (cleaved caspase-3), and blue (DAPI) fluorescence single emissions are shown, as indicated. In aged SCs cleaved caspase-3 nicely colocalized with DNA strand breaks in correspondence of nuclei and of micronuclei of myotubes in formation. White arrows point at micronuclei. Magnification: 40. Scale bar: 50 µm

Consistently, immunofluorescence analysis showed the presence of the cleaved form of caspase-3 at 72 h at both the nuclear and cytoplasmic level in the aged SCs, whereas in the young it was localized only at cytoplasmic level, with no significant differences between the two groups in terms of the number of positive cells (63.4 ± 8.9 vs 64.2 ± 12.4 respectively) (Fig. 6A). In addition, only in aged SCs caspase-3 activation was paralleled by an increased nuclear and cytoplasmic expression of myogenin, and colocalized with TUNEL in correspondence of nuclei and, moreover, of micronuclei of myotubes in formation (Fig. 6B), suggesting the involvement of caspase-3 in nuclear remodelling required during myogenesis.

**Fig. 7:**
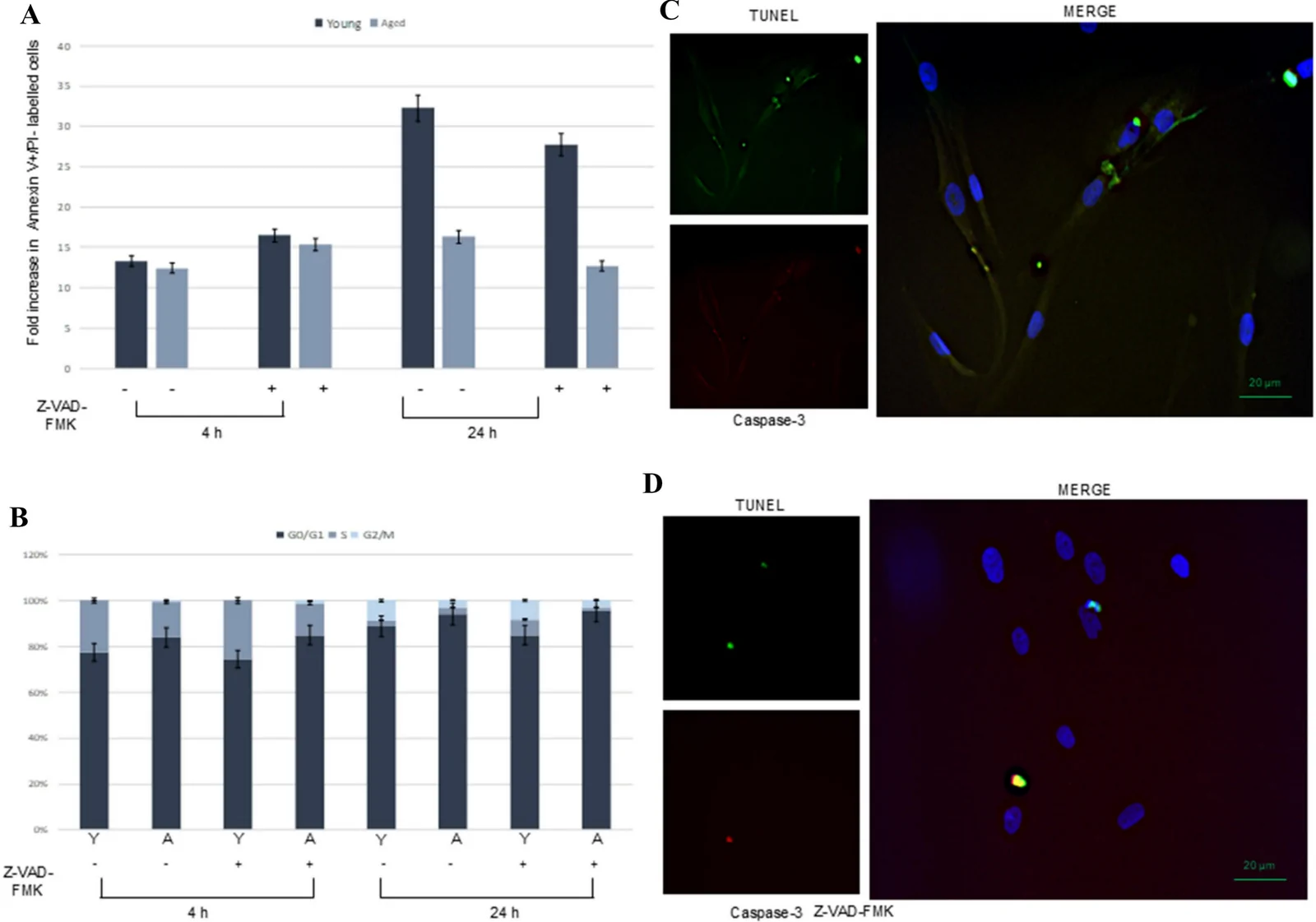
Role of the caspase enzymes. **A** Fold-increase in AnnexinV +/PI− young SCs and aged SCs prior to and after administration of a broad caspase inhibitor, z-VAD-fmk, as indicated. The data were obtained at 4 and 24 h and were normalised to the AnnexinV +/PI− cell levels without the inhibitor, as the mean ± S.E. of three independent experiments. A significant difference is seen for aged SCs versus young SCs at 4 and 24 h (p < 0.05). **B** Cell cycle distributions of young SCs and aged SCs at different time intervals of in vitro culture prior to and after administration of a broad caspase inhibitor, z-VAD-fmk, as indicated. A representative experiment of three independent experiments is shown. **C** Immunofluorescence images of cleaved caspase-3/TUNEL detection in aged SCs at 24 h of in vitro culture. Green (TUNEL), red (cleaved caspase-3), and blue (DAPI positive nuclei) fluorescence are merged in the right panel, as indicated. Of note the presence of micronuclei and myotubes in formation. Representative fields from a representative experiment of three independent experiments is shown. Magnification: 20. Scale bar: 20 µm. **D** Immunofluorescence images of cleaved caspase-3/TUNEL detection in aged SCs at 24 h of in vitro culture after administration of a broad caspase inhibitor, z-VAD-fmk. Green (TUNEL), red (cleaved caspase-3), and blue (DAPI positive nuclei) fluorescence are merged in the right panel, as indicated. Of note the dramatic reduction in micronuclei, and the absence of cleaved caspace-3 and myotubes in formation. Representative fields from a representative experiment of three independent experiments is shown. Magnification: 20. Scale bar: 20 µm

Finally, to deeply investigate the role of the caspase enzymes we treated the cell cultures with z-VAD-fmk, for up to 24 h. As already observed when inhibiting caspase-8 there was an early (4 h) rise in Annexin V + cells in the young SCs in comparison with both the untreated and the aged SCs, whereas in the aged SCs there was only a slight fold increase (Fig. 7A). In consistence with the cell cycle profile after treatment with the caspase-8 pharmacologic inhibitor, the 24 h treatment with z-VAD-fmk led to an enlarged S phase in the young and an increased G0/G1 phase in the elderly in comparison with untreated SCs (Fig. 7B). Notably, after 24 h treatment with z-VAD-fmk, the formation of micronuclei and myotubes was impaired (Fig. 7C and D) suggesting the participation of caspase enzymes in this process.

### Genes involved in cell cycle and differentiation are differentially expressed in SCs from aged and young SCs cultured under starvation (SM)

**Fig. 8:**
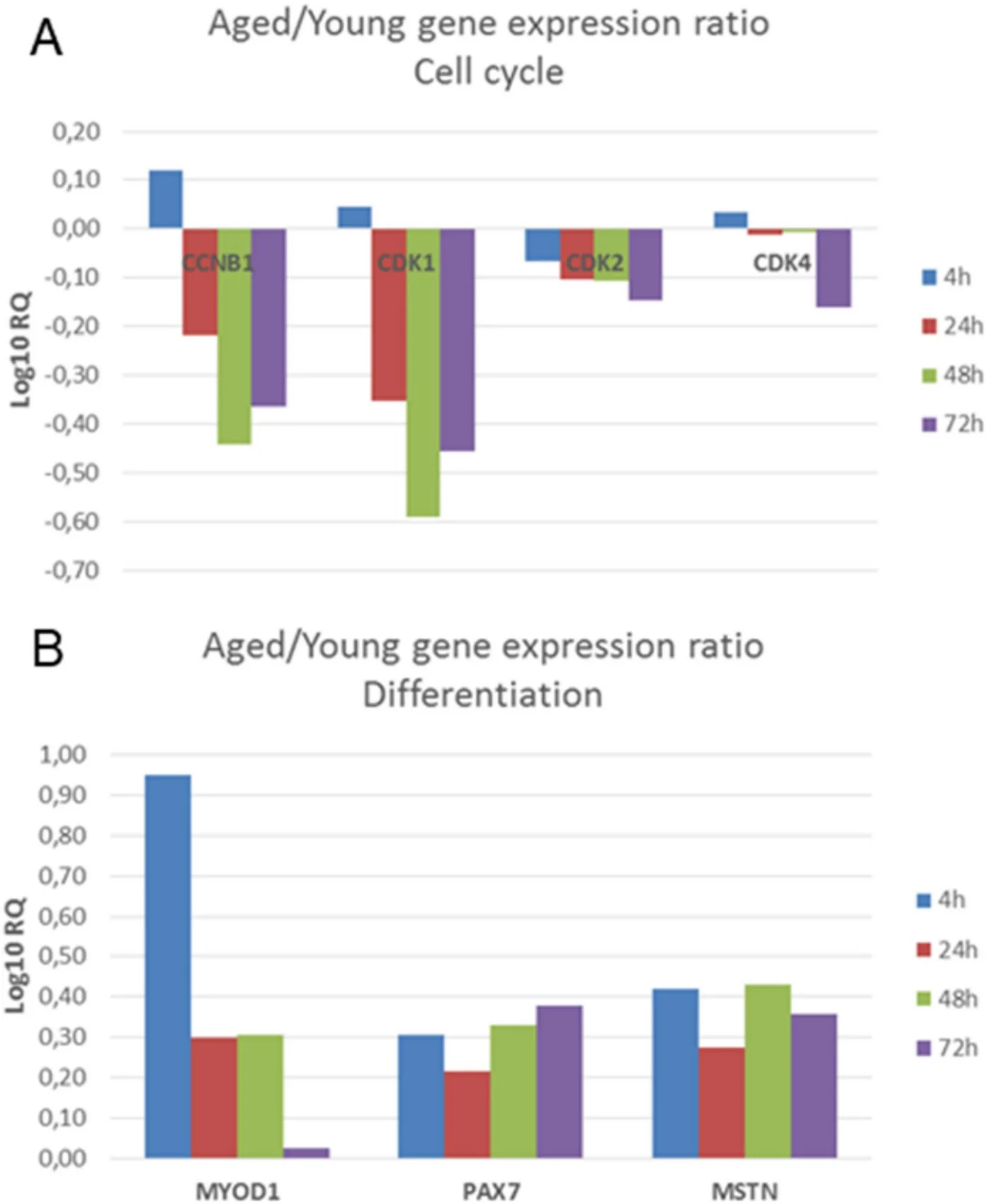
Evaluation of genes involved in cell cycle regulation and in differentiation **A** Expression levels of genes involved in cell cycle regulation, and **B** expression levels of genes representative of differentiation, analysed with RT-PCR using TaqMan low density arrays. Data from the log10 of relative quantifications of the transcripts for the target genes versus GAPDH gene expression are represented as the aged SCs to young SCs ratios, as indicated, following in vitro culture for 4–24–48 and 72 h in SM

Finally, we analysed the transcriptional profile during the early steps of the differentiation program (4–24–48–72 h in SM. Many dysregulated (up- and down-regulated) genes coding for proteins involved in cell cycle and myogenesis pathways were observed at each experimental time-point in the elderly in comparison with the young (Fig. 8). Regarding cell cycle of aged SCs cultured in SM, three genes were downregulated from 4 h up to 72 h (CDK4, CDK1 and CCNBI) and one gene was downregulated from 4 to 72 h (CDK2). CDK2 and CDK4 are involved in G1/S cell cycle checkpoints to control the commitment of eukaryotic cells to transition through the G1 phase to enter DNA synthesis S phase, while CDK1 and CCNBI are implicated in the G2/M DNA checkpoint that is responsible to arrest the cells from entering M-phase with genomic DNA damage (Fig. 8A). These observations fit well with the enlarged G0/G1 phase of the cell cycle in the elderly both in the absence (Fig. 3A) and in the presence of caspase enzymes specific inhibitors (Figs. 4C and 7B). The assessment of the genes linked to myogenesis showed an upregulation of MSTN, MyoD1 and Pax7 in SCs from aged subjects cultured in SM at all experimental time points in comparison to young ones (Fig. 8B). Noteworthy, the expression level of MyoD1 decreased with the progression of the myogenic process, while the expression levels of Pax7 and MSTN were almost the same at all experimental time points.

## Discussion

The mass, function and regenerative capacity of skeletal muscle decrease gradually with aging. It is well known that SCs are essential for recovery, supporting the repair and remodelling of muscle fibres, they are also critical to the durable support of muscle mass. Several studies have indicated the potential implication of SCs in sarcopenia. Here, we investigated the capability of human SCs from young and aged skeletal muscle to react to stress conditions. In line with our previous study (Fulle et al. 2013), aged SCs have a greater susceptibility to apoptosis in comparison to young SCs, and this trend is evident after only 4 h of cell culture (Fig. 1). Stress-induced apoptosis does not seem to involve the pathway of caspase-8 since the treatment with the specific pharmacologic inhibitor does not cause significant variations in the percentage of Annexin V +/PI− cells, whereas a dramatic rise in Annexin V +/PI− cells is evident in the young at 24 h, indicating the involvement of this initiator caspase in mediating cell survival rather than cell death in young SCs, as already demonstrated under complete medium cell culture conditions (Fulle et al. 2013).

The role of caspase-9 appears more controversial since in both age-groups the treatment with the specific pharmacologic inhibitor induces a slight decrease in the percentage of Annexin V +/PI− cells at 4 h but an increase in early apoptotic cells at 24 h especially in the elderly, confirming the non-apoptotic role of this enzyme in muscle differentiation (Murray et al. 2008). This is consistent with the microarray results showing a significant upregulation of cell proliferation-, adhesion-, growth-, development- and division-regulating genes in the presence of caspase-9 inhibition, and a parallel decrease in the expression of cell death program- and stress response-regulating genes (Fulle et al. 2013). Previous research has shown that caspase-3 activation and myotube generation are inhibited by a decrease in caspase-9 expression, demonstrating the implication in myoblast differentiation of the two genes normally involved in the mitochondrial apoptotic pathway (Ho et al. 2004; Nakanishi et al. 2007).

The different behaviour of caspase-9 is not completely understood. It is widely accepted that the promotion of differentiation and apoptosis in the myogenic lineage may employ overlapping cellular mechanisms (Fernando et al. 2002). Early initiation of the skeletal myogenic program relies heavly on caspase-3 activity. Indeed, inhibition of executioner caspase activity and homologous deletion of caspase-3 dramatically reduces myoblast fusion and myotube formation (Fernando et al. 2002). Therefore, downstream caspase activity can be exploited to promote differentiation and is shown to be regulated via a caspase-9-dependent, Bcl-xL-sensitive pathway (Fernando et al. 2002). Thus, executioner caspases act not merely as mediators of cell disassembly, but as critical determinants of myoblast fate during differentiation, as proven by Murray et al. (Murray et al. 2008). In our study, we showed the activation of caspase-3 after 72 h starvation in culture both in the young and in the elderly in which a clear co-localization with myogenin and TUNEL positive extranuclear DNA is found. The absence of DAPI positivity could be due to high levels of DNA degradation in aged cells as previously demonstrated (Lan et al. 2019). The presence of myogenin labelling in the cytoplasm with a perinuclear distribution (Fig. 6B) has been already shown in other cell models (Shiga et al. 2024) and could justify the age-related impairment in SCs differentiation (Ferri et al. 2009). Interestingly, after treatment for 24 h with z-VAD-fmk no extranuclear DNA neither myotube formation were revealed (Fig. 7B and C), highlighting the requirement of a functional caspase network in this setting. Furthermore, high-throughput screening via microfluidic cards revealed that CASP2 and CASP9 genes coding for the initiator caspases as well as CASP3 and CASP6 genes coding for the effector caspases exhibited an early upregulation trend in aged SCs whereas all the genes tested, except for CASP9 and FOXO1, were downregulated at 72 h (Fig. 5). It is worth noting that, to account for the inherent inter-individual variability typical of primary human donors, this screening was performed on a pooled sample combining equal amounts of cDNA from three (n = 3) distinct biological individuals per group. While this pooling approach provides a definitive, robustly averaged transcriptional footprint that minimizes the impact of potential individual outliers, it represents a collective population trend rather than a traditional statistically replicated cohort. Therefore, these synchronized shifts in caspase expression profile support the hypothesis of a coordinated transcriptional response to stress. A major novelty of the present study lies in the identification of a stress-specific, non-apoptotic role of caspase-3 involved in nuclear remodeling. Although a dual role for caspases in human SCs had been previously suggested under basal culture conditions (specifically regarding caspase-8 in young cells), their active participation in structural nuclear alterations under severe environmental stress was completely unknown. Here, we demonstrate for the first time that severe serum starvation—as a robust model of acute microenvironmental and metabolic stress**—**induces the formation of micronuclei containing extranuclear DNA, where active caspase-3 co-localizes with myogenin. The dramatic impairment of both micronuclei formation and myogenic differentiation following pan-caspase inhibition (z-VAD-fmk) provides clear functional evidence that caspase activity is strictly required to orchestrate nuclear remodeling under under severe starvation-induced stress. This mechanism distinctively diverges from the homeostatic pathways of spontaneous apoptosis observed during standard in vitro expansion, highlighting how environmental constraints completely reshape the functional landscape of caspase enzymes in aging muscle cells.

Nevertheless, because pan-caspase inhibition targets the cascade broadly, these findings demonstrate an absolute requirement for general caspase activity without excluding parallel contributions from other downstream effectors like caspase-6 or −7. It remains to fully elucidate the exact signaling mechanisms and specific enzymatic networks.

With regards to cell cycle the behaviour of young and aged SCs is different under stress conditions. At 4 and 24 h a greater number of cells in the elderly enter cell cycle leaving the G0/G1 phase. Also, the number of cells in S phase is significantly greater in the elderly in comparison with the young. This trend is not found in G2/M phase continuing the cell culture suggesting that aged SCs are unable to divide. The arrest in G2 of SCs is different from the arrest in G2 of differentiated cells. In these cells G2 arrest has been usually associated with the response to DNA damage to provide time for DNA repair (Sperka et al. 2012). Conversely, some authors have demonstrated that the G2 arrest that occurs in embryonic stem cells is linked with remarkable regeneration capacity (Sutcu and Ricchetti 2018). In addition, the G2 arrest could be an adaptation mechanism that cells develop in response to changes in the niche induced by aging. This hypothesis is supported by the finding that a greater expression of Pax7 and MyoD is detectable in aged SCs if compared with young SCs in our study. In fact, in SCs Pax7 and MyoD can be differently expressed during muscle regeneration (Zammit et al. 2004). In fact, while Pax7 + MyoD − cells are quiescent, Pax7 + MyoD + cells are proliferant, and Pax7 − MyoD + cells are committed to fuse to elicit polinucleated myofibers (Olguin and Olwin 2004). This is an essential process in keeping SCs reserve pool which is crucial for ongoing muscle regeneration throughout life (Motohashi and Asakura 2014). Interestingly, in the present study no hypo-diploid peak (sub-G0/G1 cell population) was observed in the cell cycle profiles of SCs. This outcome may be attributed either to the absence of characteristic DNA fragmentation at the nucleosomal level or to the incomplete progression of the apoptotic program, resulting in cellular degeneration through apo-necrosis or necrosis in cells unable to repair DNA during the G0/G1 phase of the cell cycle (Fulle et al. 2013). Previous researchers have reported that SCs are divided in two subgroups based on Pax7 expression at high- and low-level correlating with different protein levels (Rocheteau et al. 2012). Pax7-high cells show higher self-renewal potential and regeneration ability after serial engraftment into lesioned muscle than cells with low expression (Montarras et al. 2013). Furthermore, they have fewer active mitochondria and show asymmetric cell division including template DNA strand segregation that could be significant to avoid the accumulation of mutations in the stem cell daughter which will switch to quiescence (Conboy et al. 2007).

These observations suggest that under stress conditions aged SCs enter the cell cycle to exit the cell cycle before dividing and return to quiescence to reconstitute the pool of cells and try to counteract the age-related physiological decrease in their number. Indeed, a previous paper of Brack and Rando (2007) reported that in aged muscle, an early activation of SCs shifts and causes a lower proliferation and an earlier differentiation, leading to less effective regeneration (Brack and Rando 2007). This is consistent with our previous study in which we demonstrated that during aging SCs undergoing differentiation show a complex picture of compromised regenerative potential, related to the downregulation of myogenin despite the upregulation of Pax7 and MyoD (Di Filippo et al. 2016). Considering that the SM could be like the differentiation medium due to the low serum content, it can be hypothesized that the SM gives a proliferative boost that pushes SCs toward a potential differentiation that does not occur. In interpreting these findings, the caveats highlighted by Pirkmajer and Chibalin (2011) regarding the multifaceted cellular responses to serum withdrawal must be taken into account. Nonetheless, as an in vitro stress model, our data successfully isolate how primary human muscle stem cells dynamically reconfigure their apoptotic machinery and cell cycle checkpoints when exposed to extreme resource deprivation, providing key insights into the intrinsic alterations of aged satellite cells under environmental stress.

## Study limitations

Several limitations of the present study should be acknowledged. First, although the severe serum and nutrient starvation model employed herein successfully isolates how primary human satellite cells handle extreme microenvironmental constraints, it remains an in vitro system. As such, it cannot fully encapsulate the multifaceted pathophysiology, systemic signaling networks, and complex vascular interplay that characterize in vivo ischemia or tissue injury. Second, to address and mitigate the significant inter-individual biological variability inherently associated with primary human donors, our high-throughput transcriptional screening was conducted using pooled cDNA samples from three independent individuals per group. While this approach provided a definitive, robustly averaged transcriptional blueprint that effectively minimized individual outliers, it reflects a collective population trend rather than a statistically replicated, independent cohort. Finally, our experimental design focused exclusively on male donors to ensure cohort homogeneity; future investigations encompassing female donors and utilizing advanced in vivo or three-dimensional culture systems are warranted to validate the translational relevance of these caspase-mediated nuclear remodeling mechanisms during aging.

## Data Availability

The datasets analyzed in the study are available from the corresponding author upon reasonable request.

## References

[CR2] Ansari B, Coates PJ, Greenstein BD, Hall PA (1993) *In situ* end‐labelling detects DNA strand breaks in apoptosis and other physiological and pathological states. J Pathol 170:1–8. 10.1002/path.1711700102 8326456

[CR3] Beccafico S, Puglielli C, Pietrangelo T et al (2007) Age‐dependent effects on functional aspects in human satellite cells. Ann N Y Acad Sci 1100:345–352. 10.1196/annals.1395.037 17460197

[CR4] Brack AS, Muñoz-Cánoves P (2015) The ins and outs of muscle stem cell aging. Skelet Muscle 6:1. 10.1186/s13395-016-0072-z PMC4716636 26783424

[CR5] Brack AS, Rando TA (2007) Intrinsic changes and extrinsic influences of myogenic stem cell function during aging. Stem Cell Rev 3:226–237. 10.1007/s12015-007-9000-2 17917136

[CR6] Caravatta L, Sancilio S, Di Giacomo V et al (2008) PI3‐K/Akt‐dependent activation of cAMP‐response element‐binding (CREB) protein in Jurkat T leukemia cells treated with TRAIL. J Cell Physiol 214:192–200. 10.1002/jcp.21186 17579344

[CR7] Conboy MJ, Karasov AO, Rando TA (2007) High incidence of non-random template strand segregation and asymmetric fate determination in dividing stem cells and their progeny. PLoS Biol 5:e102. 10.1371/journal.pbio.0050102 17439301 PMC1852584

[CR8] Di Filippo ES, Mancinelli R, Pietrangelo T et al (2016) Myomir dysregulation and reactive oxygen species in aged human satellite cells. Biochem Biophys Res Commun 473:462–470. 10.1016/j.bbrc.2016.03.030 26975470

[CR9] Dumont NA, Wang YX, Rudnicki MA (2015) Intrinsic and extrinsic mechanisms regulating satellite cell function. Development 142:1572–1581. 10.1242/dev.114223 25922523 PMC4419274

[CR10] Ekert P, Silke J, Vaux D (1999) Caspase inhibitors. Cell Death Differ 6:1081–1086. 10.1038/sj.cdd.4400594 10578177

[CR11] Fadok VA, Voelker DR, Campbell PA et al (1992) Exposure of phosphatidylserine on the surface of apoptotic lymphocytes triggers specific recognition and removal by macrophages. J Immunol 148:2207–2216 1545126

[CR12] Fernando P, Kelly JF, Balazsi K et al (2002) Caspase 3 activity is required for skeletal muscle differentiation. Proc Natl Acad Sci USA 99:11025–11030. 10.1073/pnas.162172899 12177420 PMC123204

[CR13] Ferri P, Barbieri E, Burattini S et al (2009) Expression and subcellular localization of myogenic regulatory factors during the differentiation of skeletal muscle C2C12 myoblasts. J Cell Biochem 108:1302–1317. 10.1002/jcb.22360 19830700

[CR14] Fulle S, Didonna S, Puglielli C et al (2005) Age-dependent imbalance of the antioxidative system in human satellite cells. Exp Gerontol 40:189–197. 10.1016/j.exger.2004.11.006 15763396

[CR15] Fulle S, Centurione L, Mancinelli R et al (2012) Stem cell ageing and apoptosis. CPD 18:1694–1717. 10.2174/138161212799859657 22352749

[CR16] Fulle S, Sancilio S, Mancinelli R et al (2013) Dual role of the caspase enzymes in satellite cells from aged and young subjects. Cell Death Dis 4:e955. 10.1038/cddis.2013.472 24336075 PMC3877545

[CR17] García-Prat L, Muñoz-Cánoves P, Martinez-Vicente M (2016) Dysfunctional autophagy is a driver of muscle stem cell functional decline with aging. Autophagy 12:612–613. 10.1080/15548627.2016.1143211 26890313 PMC4835950

[CR18] Giampietro F, Sancilio S, Tiboni GM et al (2006) Levels of apoptosis in human granulosa cells seem to be comparable after therapy with a gonadotropin-releasing hormone agonist or antagonist. Fertil Steril 85:412–419. 10.1016/j.fertnstert.2005.08.020 16595220

[CR19] Ho AT, Li QH, Hakem R et al (2004) Coupling of caspase-9 to Apaf1 in response to loss of pRb or cytotoxic drugs is cell-type-specific. EMBO J 23:460–472. 10.1038/sj.emboj.7600039 14713951 PMC1271749

[CR20] Lan YY, Heather JM, Eisenhaure T et al (2019) Extranuclear DNA accumulates in aged cells and contributes to senescence and inflammation. Aging Cell 18:e12901. 10.1111/acel.12901 30706626 PMC6413746

[CR21] Montarras D, L’honoré A, Buckingham M (2013) Lying low but ready for action: the quiescent muscle satellite cell. FEBS J 280:4036–4050. 10.1111/febs.12372 23735050

[CR22] Motohashi N, Asakura A (2014) Muscle satellite cell heterogeneity and self-renewal. Front Cell Dev Bio. 10.3389/fcell.2014.00001 PMC4206996 25364710

[CR23] Murray TVA, McMahon JM, Howley BA et al (2008) A non-apoptotic role for caspase-9 in muscle differentiation. J Cell Sci 121:3786–3793. 10.1242/jcs.024547 18957517

[CR24] Nakanishi K, Dohmae N, Morishima N (2007) Endoplasmic reticulum stress increases myofiber formation *in vitro*. FASEB J 21:2994–3003. 10.1096/fj.06-6408com 17435177

[CR25] Olguin HC, Olwin BB (2004) Pax-7 up-regulation inhibits myogenesis and cell cycle progression in satellite cells: a potential mechanism for self-renewal. Dev Biol 275:375–388. 10.1016/j.ydbio.2004.08.015 15501225 PMC3322464

[CR26] Panda AC, Abdelmohsen K, Martindale JL et al (2016) Novel RNA-binding activity of MYF5 enhances *Ccnd1* / *cyclin D1* mRNA translation during myogenesis. Nucleic Acids Res 44:2393–2408. 10.1093/nar/gkw023 26819411 PMC4797292

[CR27] Pietrangelo T, Puglielli C, Mancinelli R et al (2009) Molecular basis of the myogenic profile of aged human skeletal muscle satellite cells during differentiation. Exp Gerontol 44:523–531. 10.1016/j.exger.2009.05.002 19457451

[CR28] Pirkmajer S, Chibalin AV (2011) Serum starvation: *caveat emptor*. Am J Physiol Cell Physiol 301:C272–C279. 10.1152/ajpcell.00091.2011 21613612

[CR29] Rocheteau P, Gayraud-Morel B, Siegl-Cachedenier I et al (2012) A subpopulation of adult skeletal muscle stem cells retains all template DNA strands after cell division. Cell 148:112–125. 10.1016/j.cell.2011.11.049 22265406

[CR30] Shiga S, Murakami Y, Wang Z et al (2024) An adult myogenic cell line of the Japanese fire-bellied newt *Cynops pyrrhogaster*. Sci Rep 14:30041. 10.1038/s41598-024-81899-6 39627485 PMC11614899

[CR31] Sousa-Victor P, Muñoz-Cánoves P (2016) Regenerative decline of stem cells in sarcopenia. Mol Aspects Med 50:109–117. 10.1016/j.mam.2016.02.002 26921790

[CR32] Sperka T, Wang J, Rudolph KL (2012) DNA damage checkpoints in stem cells, ageing and cancer. Nat Rev Mol Cell Biol 13:579–590. 10.1038/nrm3420 22914294

[CR33] Sutcu HH, Ricchetti M (2018) Loss of heterogeneity, quiescence, and differentiation in muscle stem cells. Stem Cell Investig 5:9–9. 10.21037/sci.2018.03.02 29780813 PMC5945914

[CR34] Zammit PS, Golding JP, Nagata Y et al (2004) Muscle satellite cells adopt divergent fates. J Cell Biol 166:347–357. 10.1083/jcb.200312007 15277541 PMC2172269

